# EKPD: a hierarchical database of eukaryotic protein kinases and protein phosphatases

**DOI:** 10.1093/nar/gkt1121

**Published:** 2013-11-08

**Authors:** Yongbo Wang, Zexian Liu, Han Cheng, Tianshun Gao, Zhicheng Pan, Qing Yang, Anyuan Guo, Yu Xue

**Affiliations:** Department of Biomedical Engineering, College of Life Science and Technology, Huazhong University of Science and Technology, Wuhan, Hubei 430074, China

## Abstract

We present here EKPD (http://ekpd.biocuckoo.org), a hierarchical database of eukaryotic protein kinases (PKs) and protein phosphatases (PPs), the key molecules responsible for the reversible phosphorylation of proteins that are involved in almost all aspects of biological processes. As extensive experimental and computational efforts have been carried out to identify PKs and PPs, an integrative resource with detailed classification and annotation information would be of great value for both experimentalists and computational biologists. In this work, we first collected 1855 PKs and 347 PPs from the scientific literature and various public databases. Based on previously established rationales, we classified all of the known PKs and PPs into a hierarchical structure with three levels, i.e. group, family and individual PK/PP. There are 10 groups with 149 families for the PKs and 10 groups with 33 families for the PPs. We constructed 139 and 27 Hidden Markov Model profiles for PK and PP families, respectively. Then we systematically characterized ∼50 000 PKs and >10 000 PPs in eukaryotes. In addition, >500 PKs and >400 PPs were computationally identified by ortholog search. Finally, the online service of the EKPD database was implemented in PHP + MySQL + JavaScript.

## INTRODUCTION

As one of the most important post-translational modifications of proteins, the reversible phosphorylation is involved in a broad spectrum of biological processes (1,2). Two types of enzymes, known as protein kinases (PKs) and protein phosphatases (PPs), are response for this reversible reaction and constitute ∼2–4% of the genes in a typical eukaryotic genome (1,3). PK is a type of well-characterized enzyme that phosphorylates proteins by chemically adding phosphate groups to specific amino acid residues, whereas PPs catalyze the dephosphorylation through the removal of ≥1 phosphate groups from the substrates (1–3). Aberrant activities of the PKs and PPs are heavily implicated in a variety of diseases, including cancers (1,4,5). The identification of the eukaryotic protein kinases (ePKs) and PPs is fundamental to a proper understanding of regulatory mechanisms of the reversible phosphorylation and will provide potential drug targets for biomedical design (6,7).

Although the concept of phosphorylation was first put forward in 1955 (8), the identification and classification of PKs has remained a great challenge. In 1995, Hanks and Hunter carried out a pilot study in which ePKs were classified into a hierarchical structure with four levels, including group, family, subfamily and individual PKs based on the conserved sequence and structural profile of the kinase (catalytic) domain (2). Subsequently, Manning *et al.* comprehensively identified 130, 454, 240 and 518 putative PKs in *Saccharomyces cerevisiae*, *Caenorhabditis elegans*, *Drosophila melanogaster* and *Homo sapiens*, respectively (1). Based on this rationale (2), these PKs were carefully curated and classified into 10 groups, 134 families and 201 subfamilies (1). However, annotation and classification of PKs at the subfamily level is time-consuming and can only be performed by hand. For example, the PKs have been clearly classified and annotated for only 11 species in the kinase.com database (1). In an effort to include more species, the Kinomer database first expanded the number of eukaryotic organisms to 52, whereas the annotation information was still only available at the group level (9). Recently, Goldberg *et al.* developed a novel software package of Kinannote, which first identified potential PKs with a Hidden Markov Model (HMM) profile in Pfam, then narrowed down the candidates by motif scoring with a position-specific scoring matrix and ultimately performed a BLAST-based classification (10). This program was used to characterize the conserved ePKs in 36 species, whereas atypical protein kinases were difficult to predict using Kinannote (10).

In contrast with PKs, the number and classification of PPs are less well understood, and most studies have focused on protein tyrosine phosphatases (PTPs) (4,11). In 2004, Alonso *et al.* systematically identified 107 putative human PTPs and classified them into four groups or classes according to their catalytic domains and also their catalytic mechanisms as well as functions (12). Based on this classification rationale, the PTP database was constructed based on 601 non-redundant PTP domains derived from 61 species (13,14). Recently, functional and structural analysis of protein serine/threonine phosphatases (PSPs) has emerged as a hot topic (15,16). The classification of PSPs is also crucial for an understanding of functional specificity and diversity (15,16). In 2008, Kerk *et al.* (3) systematically predicted and classified 150 PSPs and PTPs in *Arabidopsis thaliana*. The PPs in several other plants have also been computed and annotated (3).

In this study, 1855 PKs and 347 PPs were collected from the scientific literature and various public databases. Based on previously established rationales (1,2,12–16), we classified all of the known PKs and PPs into a three-level hierarchical structure, including group, family and single PK/PPs. There are 10 groups with 149 families for the PKs and 9 groups with 29 families for the PPs. Using HMMER (17), 139 and 27 HMM profiles were constructed for the PKs and PPs at the family level, respectively. Then we systematically characterized 49 912 PKs and 10 880 PPs in 84 eukaryotic species using the HMM profile of each family. Moreover, 521 PKs and 416 PPs were computationally identified by ortholog search. The detailed annotations from the Ensembl (18) and UniProtKB (19) databases were integrated, and the classification information was also provided. Finally, an integrative database made up of ePKs, together with the protein phosphatases database (EKPD), was developed with 50 433 PKs and 11 296 PPs. The EKPD will be regularly updated to integrate more data and information.

## CONSTRUCTION AND CONTENT

### Data collection

From the kinase.com database (1), we first obtained 1855 curated and classified PKs from *S. cerevisiae*, *C. elegans D. melanogaster*, *Mus musculus* and *H. sapiens*. The full-length protein and kinase domain sequences were directly downloaded (1). We also searched PubMed with the keyword ‘phosphatase’ and collected 347 known PPs from the scientific literature published in the period 2006–2011. The full-length PP sequences were obtained from the Ensembl (18) and UniProtKB (19) databases. The phosphatase domain information was taken from the annotations in UniProtKB. Both the kinase and phosphatase domains were further examined by searching the Pfam database (20). Moreover, we downloaded the complete proteome sets for 84 eukaryotes including 60 animals, 22 plants and 2 fungi, from Ensembl (release version 70, http://www.ensembl.org/, under the directory of ‘/pub/release-70/fasta’), EnsemblPlants (release version 16, http://plants.ensembl.org/) and EnsemblFungi (release version 16, http://fungi.ensembl.org/), respectively (18). Because a considerable number of eukaryotic proteomes had a poor annotation quality, we discarded proteins having ≥1 ‘X’ residues instead of a specific amino acid. To eliminate the redundancy, we further used ‘CD-HIT’, a tool for clustering similar sequences (21), to compare the proteins in each species separately. If multiple proteins were of 100% identity, the CD-HIT program only retained one sequence. The removed sequences were not used for any further analysis.

### Genome-wide identification of PKs and PPs

**Figure 1.**
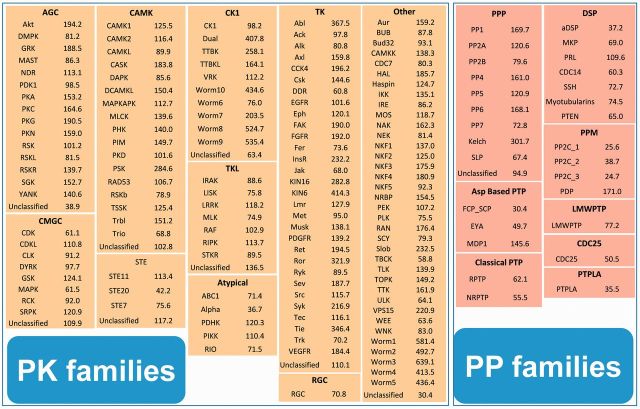
The cutoff values of the 139 and 27 HMM profiles for the PK and PP families. The hmmsearch calculates both the E-values and log-odds likelihood scores for given sequences (17). Because the E-values depend on the database size and generate inconsistent results when the database is updated, we used realistic constant log-odds likelihood scores as the threshold values.

Based on previously established rationales (1,2,12–16), we manually classified all of the curated PKs and PPs into 10 groups with 148 families and 10 groups with 33 families, respectively (Supplementary Table S1 and S2). More details on the classification of the PKs and PPs are provided in the Supplementary Results. Because the number of PKs and PPs is limited in several of the families, 139 and 27 HMM profiles were obtained for the PK and PP families, respectively. The catalytic domain sequences of the PKs and PPs were first aligned with MUSCLE (http://www.drive5.com/muscle/, version 3.8.31), an extensively used tool for multiple sequence alignment (22). HMM models were then constructed with the hmmbuild program in the HMMER 3.0 package (http://hmmer.janelia.org/) (17). Furthermore, the hmmsearch program of HMMER 3.0 (17) was separately applied to a search of all the protein sequences in 84 eukaryotes with PK and PP HMM profiles. The default parameters were adopted for the three programs. Because multiple variant peptides can originate from a single gene, here we used the Ensembl Gene ID as the unique accession to avoid any redundancy. For a given gene, only the protein with the most significant E-value was retained as the representative sequence. Again, because several similar proteins may be generated from a single gene but with different Ensembl Gene IDs, we downloaded the gene start (bp) and end (bp) information from the BioMart service of Ensembl (18) for each species. For each family, if the gene coordinates of multiple proteins were identical or overlapped, the longest one was retained. In addition, to balance the sensitivity and specificity in the prediction of new PKs and PPs, we manually selected a cutoff value for each family based on the realistic constant log-odds likelihood score in hmmsearch (17) (Figure 1). The prediction performances were also carefully evaluated subsequently (Supplementary Results and Supplementary Figure S1).

For the families without any HMM profile, we conducted orthology searches (23) to identify 521 and 416 additional PKs and PPs, respectively. As previously described (23), the strategy of reciprocal best hits was adopted by pairwise detection orthologs in the 84 eukaryotes. The blastall program in the BLAST package was used (24). All of these HMM profiles can be freely downloaded at http://ekpd.biocuckoo.org/faq.php.

### A landscape of ePKs and PPs

**Figure 2.**
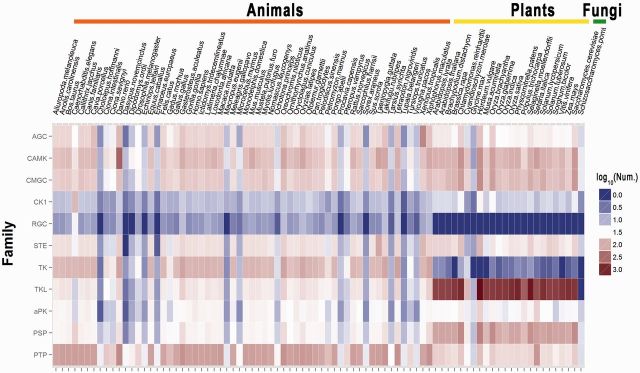
The heatmap of the classifications and the numbers of proteins for several major groups. Nine major groups of the PKs shown. For the PPs, the groups of PSPs and PTPs were visualized. The numbers of the PKs or PPs is commonly different across species. For example, RGC kinases have been exclusively detected in animals and not plants. Also, TKL kinases are only present in a small proportion of the animal PKs (8.2%), but are widely represented in plants (60.4%).

In total, 50 433 PKs and 11 296 PPs from 84 eukaryotic species were identified, with an average of 600.4 PKs and 134.5 PPs per organism (Supplementary Table S1 and S2). Although there are 395.5 PKs on average per animal, the average number of plant PKs is 3-fold (1202.0) higher than animals (Supplementary Table S1). A heatmap of the classifications and identification patterns for several of the major PK and PP groups was visualized using the ggplot2 program (http://had.co.nz/ggplot2/) in the R package (http://www.r-project.org/) (25) (Figure 2). From the results, the numbers of animal or plant PKs in the same group or family can differ greatly (Figure 2). For example, we identified 310 RGC PKs in 60 animals with an average number of 5.2 per species, whereas no RGC kinases were detected in 22 plants (Supplementary Table S1). Also, there are 66.1 TK kinases on average per animal, whereas only 2.7 TK kinases on average were detected per plant (Supplementary Table S1). In addition, only a small proportion of PKs are TKL kinases (8.2%) in animals, whereas up to 60.4% of plant PKs were classified in the TKL group (Supplementary Table S1). This result is consistent with previous analyses, which have shown that TKL kinases predominantly occur in plants (26). The average numbers of PPs are moderately different, as they were 126.0 and 166.3 per animal and plant organisms, respectively (Supplementary Table S2).

PKs and PPs are typically multidomain proteins containing a variety of other protein domains in addition to the catalytic domain. To identify the proteins domains that co-occur with the kinase and phosphatase catalytic domains, the distribution and diversity of the Pfam domain in the PKs (Supplementary Table S3) and PPs (Supplementary Table S4) were statistically analysed using a the hypergeometric test (27) (Supplementary Methods, *P* < 10^−^^8^). Although a number of Pfam domains are preferentially over- or under-represented in both PKs and PPs, more domains are exclusively enriched or deprived in PKs or PPs (Supplementary Table S3 and S4). For example, SH2 (PF00017), which acts as a type of phosphotyrosine interacting domain (28), is significantly enriched in both the PKs and PPs (Supplementary Table S3 and S4). Meanwhile, the F-box domain (PF00646), which is found in specific proteins that recruit substrates for ubiquitination and proteasomal degradation (29), is significantly under-represented in both PKs and PPs (Supplementary Table S3 and S4). However, another type of phosphotyrosine-binding domain, PTB (PF08416) (28) is only significantly over-represented in PPs (Supplementary Table S4), whereas the SH3 domain (PF00018) (30), which is responsible for protein–protein interactions, is significantly enriched only in the PKs (Supplementary Table S3). Furthermore, we statistically compared the different preferences of Pfam the domains in PKs (Supplementary Table S5) and PPs (Supplementary Table S6) in animals and plants using Yates' chi-squared (χ^2^) test (27) (Supplementary Methods, *P* < 10^−^^8^). Interestingly, the SH2 domain occurs preferentially in both animal PKs and PPs compared with plants (Supplementary Table S5), whereas the SH3 domain preferentially occurs only in animal PKs (Supplementary Table S6).

## USAGE

**Figure 3.**
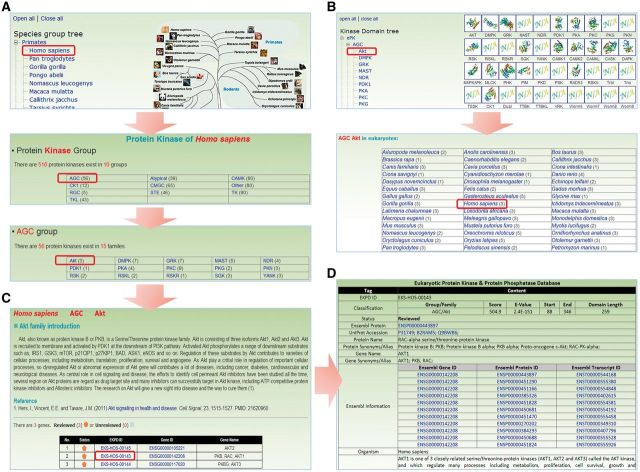
The browse option of EKPD. We provided two approaches for browsing the database: (**A**) By species. (**B**) By classifications. (**C**) For a PK or PP family, a brief description and the associated members are shown. (**D**) Detailed information on human AKT1.

The EKPD database was developed so as to be operable in an easy-to-use manner. Here we provide human protein kinase B (PKB or AKT1) as an example to illustrate the effective usage of EKPD. To make it easy to look through the data in EKPD, two approaches were implemented for the browse option: by species or by classification (Figure 3). In the option of ‘browse by species’, the left tree represents the Ensembl taxonomy categories, including primates, rodents, laurasiatheria and so on, whereas the right tree represents the phylogenetic relationship of the eukaryotic species in Ensembl (18) (Figure 3A). By clicking on the ‘*Homo sapiens*’ button, the PK and PP groups in *H. sapiens* can be viewed (Figure 3A). As the Akt family belongs to the AGC group, users can click on the ‘AGC’ button to view the family information (Figure 3A). Also, EKPD can be further browsed by classification (Figure 3B). The left tree represents the hierarchical classification, whereas a representative 3D structure of the catalytic domain was taken from the PDB (31) and presented on the right for each PK or PP family, if available (Figure 3B). Users can click on the ‘Akt’ button to visualize the family information across 70 eukaryotes (Figure 3B). By either clicking on the ‘Akt’ button in the AGC group page (Figure 3A) or the ‘*Homo sapiens*’ button in the Akt page (Figure 3B), the members in human Akt family can be viewed, while a brief description of Akt functions and regulatory roles is available (Figure 3C). To organize the database, we used EKPD IDs for the PKs (EKS-) and PPs (EPS-), respectively. The Ensembl Gene ID was adopted as the secondary accession (Figure 3C). The users can click on the ‘EKS-HOS-00143’ button to view the detailed information of human AKT1 (Figure 3D). More detailed descriptions of the search and advance options in EKPD were also presented (Supplementary Results and Supplementary Figure S2).

## DISCUSSION

The identification and classification of PKs and PPs are fundamental for characterizing the regulatory roles of phosphorylation and dephosphorylation (1–3), predicting the kinase-specific phosphorylation sites in proteins (32), modeling potential phosphorylation networks (33), detecting disease- or cancer-associated mutations (34,35) and providing potential targets for drug design (6,7). A comprehensive data resource with detailed annotation and classification information would be of great benefit for further studies.

A number of public databases have been previously developed, such as KinG (36), Protein kinase resource (PKR) (37), Kinase.com (1), Kinomer (9), KinMutBase (34) and MoKCa (35), for the PKs, and the PTP database (13,14) and PhosphaBase (38) for the PPs. The KinG database contains the PKs from five eukaryotic species *S. cerevisiae*, *C. elegans*, *D. melanogaster*, *H. sapiens* and *A. thaliana* (36), whereas PKR contains PK information for eight species (37). The most well-annotated resource is Kinase.com, which has classified PKs in 11 eukaryotes at the subfamily level (1). However, such an annotation is labor-intensive and largely dependent on manual curation. In this regard, the Kinomer classified PKs of 52 eukaryotic species at the group level (9). With identified PKs in human, KinMutBase (34) and MoKCa (35) were developed to contain disease- or cancer-associated mutations in PKs, respectively. For PPs, the PTP database contains the known information for PTPs across 61 species, whereas the PSPs have not been integrated (13,14). In addition, PhosphaBase collected >2800 known PPs from the scientific literature and public databases for 345 species, with an average number of eight PPs per organism (38). Thus, this data set is evidently far from being integrative.

**Figure 4.**
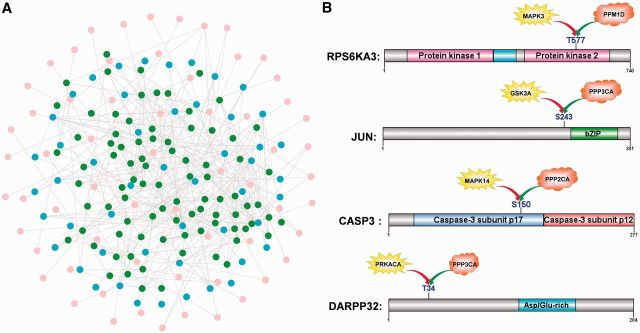
The kinase-phosphatase relations via common substrates. (**A**) A human kinase-phosphatase network was reconstructed with 62 PKs (pink), 50 PPs (blue) and 87 common substrates (green). (**B**) Cases of site-specific kinase-phosphatase relations. For example, the phosphorylation and dephosphorylation of T577 regulates the kinase activity of RPS6KA3 (Supplementary Table S7).

In eukaryotes, a protein substrate is phosphorylated by PKs and dephosphorylated by PPs (33,39). The identification of kinase-phosphatase relations via their common substrates is helpful for understanding the reversible regulatory process of phosphorylation. Due to data limitations, we only analysed the kinase-phosphatase relations in *H. sapiens*. From the Phospho.ELM database (version 9.0), we obtained 2436 human phosphorylation sites modified by known PKs (40). Also, we took 317 dephosphorylation sites with known regulatory PPs from human DEPhOsphorylation Database (DEPOD) (39). With the two data sets, we detected 87 common substrates with 146 sites that had both upstream regulatory PKs and PPs (Supplementary Table S7). Based on the identified site-specific kinase-substrate and phosphatase-substrate relations, we reconstructed a human kinase-phosphatase network, containing 62 PKs, 50 PPs and 87 common substrates (Figure 4A). In particular, there were 31 PKs and 5 PPs in the common substrates. The intensive interactions between PKs and PPs through common substrates suggest that the phosphorylation regulation is highly specific and dynamic. For example, human p90 ribosomal protein S6 kinase alpha-3 (RPS6KA3) is modified by MAPK3 at T577, which can be dephosphorylated by protein phosphatase 2C delta (PPM1D) to reduce the kinase activity (41) (Figure 4B and Supplementary Table S7). Furthermore, the S243 of transcription factor AP-1/c-Jun (JUN) is phosphorylated by GSK3A and dephosphorylated by PPP3CA, whereas the dephosphorylation regulates the c-Jun/Sp1 interaction (42,43) (Figure 4B and Supplementary Table S7).

Taken together, our database and the associated results provide a useful resource for further analysis, although improvement is still needed. For example, the specific nomenclatures for plant groups or families should be adopted, once a greater number of PKs and PPs have been experimentally identified in plants. Also, the classification and annotation information is not yet optimal for several species, as certain genomes are poorly annotated and have various types of errors. In this regard, the EKPD database will be continuously updated and improved as the current proteome sets are updated and more species are made available.

## SUPPLEMENTARY DATA

Supplementary Data are available at NAR Online, including [1, 2, 12–16, 18, 20, 27].

## FUNDING

Funding for open access charge: National Basic Research Program (973 project) [2012CB910101, 2013CB933903 and 2012FY112900]; Natural Science Foundation of China [31171263 and 81272578]; International Science & Technology Cooperation Program of China [0S2013ZR0003].

*Conflict of interest statement*. None declared.
